# Knowledge on the action to be taken and recognition of symptoms of stroke in a community: findings from the May Measurement Month 2017 blood pressure screening Programme in Malaysia

**DOI:** 10.1186/s12889-019-7922-7

**Published:** 2019-11-29

**Authors:** SiewMooi Ching, Yook Chin Chia, Bee Nah Chew, Man Jun Soo, Hooi Min Lim, Wan Aliaa Wan Sulaiman, Fan Kee Hoo, Mar Lar Saw, Azlina Ishak, Tharmalingam Palanivelu, Nadarajan Caruppaiya, Navin Kumar Devaraj

**Affiliations:** 1 Department of Family Medicine, Faculty of Medicine and Health Sciences, Universiti Putra Malaysia, Serdang, Selangor, Malaysia; 2 Malaysian Research Institute on Ageing (MyAgeing), Universiti Putra Malaysia, Serdang, Selangor, Malaysia; 3 Department of Medical Sciences, School of Healthcare and Medical Sciences, Sunway University, Subang Jaya, Selangor Malaysia; 4 Staff & Student Health Unit, University Malaya Medical Centre, Kuala Lumpur, Malaysia; 5 Faculty of Medicine and Health Sciences, Universiti Putra Malaysia, Serdang, Selangor Malaysia; 6 Department of Primary Care Medicine, University Malaya Medical Center, University of Malaya, Kuala Lumpur, Malaysia; 7 Department of Medicine, Faculty of Medicine and Health Sciences, Universiti Putra Malaysia, Serdang,Selangor, Malaysia; 8 Department of Family Medicine, School of Medical Sciences, Health Campus, UniversitiSains Malaysia, Kubang Kerian, Kelantan Malaysia; 9 Department of Medicine, Hospital Sultan Abdul Halim Kuala, Sg.Petani, Kedah, Malaysia; 10 Faculty of Medicine, Asian Institute of Medicine, Science and Technology (AIMST) University, Semeling, Sg.Petani, Kedah Malaysia

**Keywords:** Knowledge, Practice, Stroke, May measurement month, Malaysia, Symptoms

## Abstract

**Background:**

Information regarding the knowledge on the action to be taken during stroke and good recognition of stroke symptoms is mandatory in helping to plan out educational strategies to deliver health education to the community.

**Methods:**

This is a cross-sectional study of adults aged 18 and above attending a blood pressure screening program in community in conjunction with May Measurement Month 2017 in Malaysia. A structured self-administered questionnaire was given to the participants who gave verbal consent. Data analysis was done using SPSS v. 23 and multiple logistic regression was used to identify the determinants of knowledge on actions to be taken during stroke and recognition of stroke symptoms.

**Results:**

Out of 4096 respondents, 82.9–92.1% of them able to recognise the common stroke symptoms. and 74.2% of the study respondents will go to hospital within 4.5 h of stroke onset. According to binomial logistic regression analyses, adults aged 45 years old and above (OR 1.39 95%CI 1.01–1.92), being Malay (OR 1.74, 95% CI 1.27–2.40), being non-smokers (OR = 2.491, 95% CI: 1.64–3.78), hypertensives (OR: 1.57, 95% CI: 1.02–2.42)and diabetics (OR: 2.54, 95% CI:1.38–4.69) are determinants of right actions to be taken during stroke. Meanwhile, respondents aged 45 years old and older (OR = 1.68, 95% CI: 1.39–2.03), being Malay (OR = 1.49, 95% CI: 1.24–1.79), hypertensive (OR = 1.32, 95% CI: 1.04–1.66) and those who had a previous history of stroke (OR = 2.25, 95% CI: 1.01–5.00) are determinants of good recognition of stroke symptoms.

**Conclusions:**

The overall knowledge of stroke in our study population was good. Older age, being Malay, non-smokers, hypertensives and diabetics are determinants of right actions to be taken during stroke. Meanwhile, older age, being Malay, hypertensive and those who had a previous history of stroke are determinants of good recognition of stroke symptoms.

## Background

Stroke is a clinical syndrome characterized by rapidly developing clinical syndromes and/or signs of focal, and at times global, loss of cerebral function, with symptoms lasting more than 24 h or leading to death, with no apparent cause other than that of vascular origin [1]. Stroke has been the second leading cause of death after ischemic heart disease in 2016, which causes at least 15 million deaths worldwide [2]. Comparable to the data from World Health Organization, it was reported that as every hour passes by, there will be 6 new cases of stroke in Malaysia [3]. Furthermore, in Malaysia, stroke was ranked third as the leading cause of death in male after ischemic heart disease and pneumonia, and ranked second in female as the leading cause of death after ischemic heart disease [4].

Despite extensive studies devoted to the management and prevention of stroke, the global burden of stroke would not be reduced without efforts targeting good public knowledge and practice [5]. A review has shown that there is a huge gap in terms of the knowledge of stroke between developing and developed countries, in which poor recognition of stroke symptoms are more of a problem in developing countries [6]. Besides that, it was stated that certain stroke patients in developing countries would prefer to go to their personal general practitioners or seek for alternative treatment instead of going to emergency department immediately [7–9]. The prime importance of promptly going to the hospital is based on the fact that the golden hour of 4.5 h after the onset of stroke will enable the administration of rTPA to the patients which may cure the stroke altogether [1]. Notably in Malaysia, there are very few studies that examine the level of knowledge and practice of stroke [6]

Lack of knowledge on the actions to be taken during stroke and recognition of stroke symptoms often result in a delay in hospital presentation and early intervention which may lead to prolonged hospital stay which will then subsequently lead to an increase of stroke-related morbidities and mortality. This has inevitably caused significant impact towards patient’s quality of life, functional ability and interpersonal relationships [6]. Therefore, baseline information regarding the knowledge on the actions to be taken during stroke and recognition of stroke symptoms in the community is important in planning out educational strategies to deliver health education to the community [10]. This study aims to study the level of knowledge on the actions to be taken during stroke and recognition of stroke symptoms and its associated factors among the general public in Malaysia.

## Methodology

### Study design

This was a cross-sectional nationwide community blood pressure screening programme of adults aged 18 years and above. The screening programme was carried out at various clinics, hospitals and community centres and also during family day events and charity runs from April to May 2017 involving a total of 42 centres in Malaysia. The medical professionals consisting of 64 doctors helped in the data collection [11].

The Malaysian Society of Hypertension also collaborated with the Department of Primary Care Medicine, University Malaya Medical Center (UMMC) in conducting various health-related activities such as exhibitions, cooking demonstrations of healthy food, public educational talks with concurrent blood pressure screening of the attendees during that periods.

Along with the blood pressure screening, a self-administered questionnaire was provided to the participants assessing their knowledge on the actions to be taken during stroke and recognition of stroke symptoms after obtaining their verbal consent.

### Inclusion criteria

The inclusion criteria for the study were adults aged 18 years and above.

### Sample size calculation

As this is a public screening programme, the sample size was not calculated. A local study had reported that the prevalence of satisfactory knowledge on recognizing warning signs was 35%, while for risk factors of stroke was 29% [12]. Based on this value, the estimated sample size would be 1376 with a power of 99.99%, α value of 5% and a 95% confidence interval (CI).

### Sampling method

A universal sampling method was used to recruit participants where all participants who consented verbally to participate were recruited into this study.

### Data collection instrument

The questionnaire is self-administered and comprising two sections. The first section explored the sociodemographic characteristics of the participants and their relevant past medical history. The second section explored participant’s knowledge on the actions to be taken during stroke and recognition of stroke symptoms.

We derived the 5 symptoms based on the acronym “F.A.S.T” (facial weakness, arm weakness speech problem and time) recommended by the American Stroke Association to help the public recognise stroke early and seek treatment early too [13]. We also included another two questions on the common symptoms of “paralysis of one side of the body” and numbness or tingling sensation of one side of the body because these symptoms are commonly seen in middle cerebral artery occlusion. We didn’t ask other symptoms particularly homonymous hemianopia as participants may not recognize it [1].

The questionnaire was developed by the authors based mainly on the local clinical practice guideline on the management of ischaemic stroke [1]. Content validity was done by 5 authors in which included 3 Family Medicine Specialists and 2 senior consultant neurologists. The authors critically appraise all the items to verify its adequacy,clarity, appropriateness and meaningfulness. Modification of the items was done based on these experts’ suggestions and comments. The modification that had been done were:

1. The experts suggested to add the don’t know option for question 2.3, 2.5 and 2.6 where initially only 2 answer options which was Yes and No (Can a person have stroke more than once? Which are the clinical features of stroke?, What would you do if you suffer from a stroke?)
2. “Numbness tingling sensation of one side of the body” question was also added based on the experts’ suggestion.
3. “Go to hospital within same day” question was also added based on the expert’s suggestion.

There was no psychometric testing done and the format of the questions were closed ended. Malay and English language were the language of instruments used in the questionnaires. The authors initially produced the questions in English and then translated it back into the Malay language. Forward and backward translation was done during the translation process.

### Study procedure

Participants of this study were given a self-administered questionnaire as detailed above before blood pressure measurements were carried out. Participants that had problem in answering the questions due to vision problems were helped by the on-site researcher in filling-up the questionnaire. Data on sociodemographic and knowledge on the actions to be taken during stroke and recognition of stroke symptoms were collected concurrently during the blood pressure screening programme by trained researcher who were mainly medical professionals. Respondents were allowed to answer more than one option for question on “what types of action to be taken during stroke”.

Three blood pressure measurements were taken following the standard procedure using digital blood pressure devices (Omron model number HEM-7120, RossMax model and Buerer model no BM28). The first reading of blood pressure was taken after 5 minutes of rest, while the second and third reading of blood pressure was taken after 1 minute of rest each.

A pamphlet with information on the risk factors and complications of hypertension, importance of yearly blood pressure measurement together with recommended lifestyle modifications hypertension prevention(i.e. practicing a low salt diet, increasing physical activity and smoking cessation) was given to all respondents. The on-site researchers also delivered health talk on the causes, complications and prevention of hypertension at the end of the screening programme at some centres especially during family events or charity run. The complication of hypertension was just mentioned as heart attacks, stroke, heart failure, kidney failure. No further details were given about stroke during the health talks. Similarly, no further information was given on stroke risk factors and actions that needs to be taken in the event of a stroke. In those who were noted to have elevated blood pressure, they were further advised to visit the nearest healthcare facilities to verify their elevated blood pressure status after an interval of 2–4 weeks.

### Operational definition

Good knowledge on the actions to be taken during stroke is defined as those who answered “go to hospital within 4.5 hours” and/or “go to hospital within same day” to the question of “What would you do if you suffer from a stroke?”. Good recognition of stroke symptoms was defined as those who were able to identify all 5 symptoms of stroke correctly.

### Data analysis

Statistical Package for Social Sciences (SPSS) version 23.0 was used for data analysis. Descriptive analysis was used to describe the characteristics of the respondents in terms of frequencies, percentages, means, and standard deviations. In this study, we used Chi-square test to identify the associations between ‘recognition of stroke symptoms’ with the independent factors as well as the association between knowledge of the action to be taken in the event of a stroke and the independent factors. However, inferential statistics was not used for other variables such as ‘organ affected in stroke’, effect of stroke on ‘ADLs’, ‘is stroke preventable’ and ‘can a person have a stroke more than once’.

All variables with the *p* value < 0.25 in the univariate analysis (chi square test) as well as additionally clinically significant variables were entered into the binomial logistic regression analysis. Binomial logistic regression analysis was used to identify the determinants of good recognition of stroke symptoms and right action needed to be taken during the event of a stroke. The dependent variable was recognition of stroke symptoms and actions to be taken during stroke. The independent variables were age, gender, ethnicity, education level, employment status, marital status, smoking status, alcohol consumption, hypertensive status, diabetes status, personal history of heart attack and personal history of stroke. Variables that a *p* value of < 0.05 was taken as being statistically significant in the final logistic regression model.

## Results

### Socio-demographic characteristics

A total of 4096 respondents were recruited into this study which exceeded the estimated sample size. Table 1 demonstrated the sociodemographic characteristics of the study respondents. Median age of the study respondents was 45.0 (34) years old. 59.5% (*n* = 2429) of the study respondents were female. Our study respondents were predominantly Malay Muslims (49.6%) with majority of them attaining at least secondary level of education (42.5% attained secondary level of education and 42.9% attained tertiary level of education). 54.1% of the respondents were married. Only 7.2% of the respondents smoked but there were 69.2% of them who consumed alcohol at least once a week.

**Table 1:** Sociodemographic Characteristics of Study Respondents (*n* = 4096)

Variable	Frequency N (%)	Median (IQR)
Age, years		45.0(34)
Gender		
Male	1656 (40.5)	
Female	2429 (59.5)	
Missing data	11	
Ethnicity		
Malay	1567 (49.6)	
Chinese	831 (26.3)	
Indian	683 (21.6)	
Others	80 (2.5)	
Missing data	935	
Education Level		
No formal education	21 (3.3)	
Primary school	72 (11.3)	
Secondary school	270 (42.5)	
College/University	273 (42.9)	
Missing data	3460	
Employment status		
Unemployed	156 (34.5)	
Employed	296 (65.5)	
Missing data	3644	
Marital status		
Married	379 (54.1)	
Single	322 (45.9)	
Missing data	3395	
Smoking		
Yes	203 (7.2)	
No	2594 (92.8)	
Missing data	1299	
Alcohol		
Never	860 (30.7)	
Once a week	1935 (69.2)	
Regularly	2 (0.1)	
Missing data	1299	
Personal History of Heart Attack		
Yes	134 (4.8)	
No	2663 (95.2)	
Missing data	1299	
Personal History of Stroke		
Yes	64 (2.3)	
No	2733 (97.7)	
Missing data	1299	
Diabetes		
Yes	444 (15.9)	
No	2353 (84.1)	
Missing data	1299	
Hypertension		
Yes	894 (23.8)	
No	2868 (76.2)	
Missing data	334	

Missing data is only expressed in frequency

### Knowledge of stroke

Table 2 shows the responses towards question on the knowledge on the actions to be taken during stroke and recognition of stroke symptoms among study respondents. 63% of them knew that the brain is the organ affected by stroke. Among those who answered wrongly, they identified heart (16%), kidney (2.1%), lung (1.3%) and liver (0.5%) as the organs in which stroke occurs. 17.1% of them did not know which is the organ affected by stroke. Majority of the respondents were able to identify facial symmetry (88.0%), sudden difficulty in speaking/slurred speech (92.1%), weakness of one side of body (90.3%), paralysis of one side of body (90.6%) and numbness tingling sensation of one side of the body (82.9%) as symptoms of stroke. 83.4% of the respondents answered that stroke is preventable and 82.2% of them agree that stroke can occur more than once in the same individual. More than 60% of the study respondents agree that stroke can affect their daily life.

**Table 2:** Responses of Study Respondents to the Questions on Knowledge of Stroke (*n* = 4096)

Variables	Yes, n (%)	No, n (%)
Which organ of the body is affected by stroke?		
Brain	2484 (63.0)	–
Kidney	82 (2.0)	–
Lung	51 (1.3)	–
Heart	631 (16.0)	–
Liver	19 (0.5)	–
I don’t know	673 (17.1)	–
Which are the important symptoms of stroke?		
Facial Asymmetry	3593 (88.0)	489 (12.0)
Sudden difficulty in speaking / slurred speech	2765 (92.1)	325 (7.9)
Weakness of one side of the body	3685 (90.3)	397 (9.7)
Paralysis of one side of the body	3698 (90.6)	385 (9.4)
Numbness tingling sensation of one side of the body	3382 (82.9)	697 (17.1)
Is stroke preventable?	3410 (83.4)	678 (16.6)
Can a person have stroke more than once?	3359 (82.2)	728 (17.8)
Does stroke have any effect on any of these daily activities?		
Dressing yourself	3023 (74.1)	1059 (25.9)
Going to the toilet without help	2893 (70.9)	1188 (29.1)
Being able to work	2569 (63.0)	1510 (37.0)
Driving a car	2496 (61.2)	1584 (38.8)
What would you do if you suffer from a stroke? *		
Go to hospital within 4.5 h	3017 (74.2)	1047 (25.8)
Go to hospital within same day	3343 (82.1)	727 (17.9)
Visit alternative health care providers	1283 (31.6)	2774 (68.4)
Combination of hospital and tradition	1908 (47.0)	2154 (53.0)
Seek spiritual healing	2196 (54.0)	1868 (46.0)

* Respondents were allowed to answer more than one option for question on: “what types of action to be taken during stroke”

In regards of the number of respondents who identified the symptoms of stroke correctly, 74.3% of the study respondents successfully identified all 5 symptoms of stroke while about 4% of them weren’t able to identify even one of the symptoms of stroke.

In terms of knowledge on the actions to be taken during stroke, 74.2% (*n* = 3017) of respondents answered that they will go to hospital within 4.5 h of the onset of stroke and 82.1% (*n* = 3343) will go to the hospital within the same day of the onset of stroke. However, it was found that 31.6% of the respondents will also seek for alternative health care providers, 47.0% of them will seek a combination of hospital and alternative treatments and 54% of them will opt for spiritual healing.

Factors Associated with Action to be taken during Stroke and Recognition of Stroke Symptoms.

Table 3 displays the association between socio-demographic characteristics and past medical history with the actions to be taken during stroke and recognition of stroke symptoms. Chi-Square analysis showed that age, gender, ethnicity, smoking status, hypertension, diabetes and past history of heart attack had a significant association with action to be taken during stroke among the study respondents. Chi-Square analysis also indicated that age, ethnicity and hypertension had a significant association with good recognition of stroke symptoms among the study respondents.

**Table 3:** Association between Sociodemographic Characteristics and Past Medical History with knowledge on the action to be taken during stroke and Recognition of Stroke Symptoms

Variables	Actions to be taken during stroke			Recognition of Stroke Symptoms		
	Poor Practice, n (%)	Good Practice, n (%)	*p*-value	Poor Recognition, n (%)	Good Recognition, n (%)	*p*-value
Age			< 0.001			0.001
< 45	614 (31.6)	1328 (68.4)		344 (17.6)	1613 (82.4)	
≥ 45	433 (20.4)	1689 (79.6)		292 (13.7)	1839 (86.3)	
Gender			0.002			0.056
Male	383 (23.3)	1264 (76.7)		279 (16.9)	1373 (83.1)	
Female	663 (27.6)	1743 (72.4)		356 (14.7)	2069 (85.3)	
Ethnicity			0.001			< 0.001
Malay	422 (27.0)	1143(73.0)		184 (11.7)	1383 (88.3)	
Non-Malay	348 (22.0)	1237 (78.0)		284 (17.9)	1307 (82.1)	
Marital Status			0.762			0.644
Single	99 (31.4)	216 (68.6)		57 (17.8)	263 (82.2)	
Married	119 (32.5)	247 (67.5)		62 (16.5)	314 (83.5)	
Employment status			0.254			0.825
Unemployed	39 (25.8)	112 (74.2)		28 (18.1)	127 (81.9)	
Employed	90 (31.0)	200 (69.0)		51 (17.2)	245 (82.8)	
Education Level			0.048			0.126
None/Primary	22 (23.7)	71 (76.3)		21 (22.6)	72 (77.4)	
Secondary/Tertiary missing	179 (34.1)	346 (65.9)		87 (16.1)	453 (83.9)	
Smoking			0.004			0.941
Yes	71 (31.8)	152 (68.2)		33 (14.7)	192 (85.3)	
No	597 (23.3)	1963 (76.7)		372 (14.5)	2196 (85.5)	
Alcohol			0.426			0.153
Yes	0 (0.0)	2 (100.0)		1 (50.0)	1 (50.0)	
No	671 (24.1)	2114 (75.9)		404 (14.5)	2391 (85.5)	
Hypertension			0.006			0.002
Yes	48 (5.4)	846 (94.6)		15 (10.9)	122 (89.1)	
No	234 (8.2)	2634 (91.8)		68 (16.4)	346 (83.6)	
Diabetes			< 0.001			0.219
Yes	57 (14.0)	350 (86.0)		51 (12.5)	357 (87.5)	
No	614 (25.8)	1766 (74.2)		354 (14.8)	2035 (85.2_	
Past History of Heart Attack			0.016			0.356
Yes	21 (15.4)	115 (84.6)		16 (11.8)	120 (88.2)	
No	650 (24.5)	2001 (75.5)		389 (14.6)	2272 (84.4)	
Past History of Stroke			0.476			0.125
Yes	13 (20.3)	51 (79.7)		5 (7.8)	59 (92.2)	
No	658 (24.2)	2065 (75.8)		400 (14.6)	2333 (85.4)	

Table 4 shows the binomial logistic regression analysis of the associated factors of the actions to be taken during stroke and good recognition of stroke symptoms. In terms of practice of stroke, it was found that study respondents aged 45 years and above are at 1.39 times (95% CI: 1.01–1.92, *p* = 0.043) more likely to have good practice of stroke compared to those aged less than 45 years old. Being a Malay is at 1.74 times higher odds (95% CI: 1.27–2.40, *p* = 0.001) of having a good knowledge of the actions to be taken during stroke. Those study respondents who did not smoke are 2.49 times more likely to have good knowledge of the actions to be taken during stroke in contrast to those who smoke (95% CI: 1.64–3.78, *p* < 0.001). Those who have hypertension are 1.57 times (95% CI: 1.02–2.42, *p* = 0.042) more likely to have good knowledge of the actions to be taken during stroke as compared to those who are not hypertensive. Furthermore, those who are diabetic are also at 2.54 odds more likely to have good knowledge of the actions to be taken during stroke as compared to non-diabetics (OR = 2.54, 95% CI: 1.38–4.69, *p* = 0.003).

**Table 4:** Multiple Logistic Regression of the Associated Factors for Action to be taken during stroke and Good Recognition of Stroke Symptoms

Variables	Actions to be taken during stroke			Good Recognition of Stroke Symptoms		
	Adjusted OR	95% CI (Lower, upper)	*P* value	Adjusted OR	95% CI (Lower, upper)	*P* value
Age		1.01–1.92	0.043		1.39–2.03	< 0.001
< 45	1			1		
≥ 45	1.39			1.68		
Ethnicity		1.27–2.40	0.001		1.24–1.79	< 0.001
Malay	1.74			1.49		
Non-Malay	1			1		
Smoking Status		1.64–3.78	< 0.001		0.90–1.67	0.196
Yes	1			1		
No	2.49			1.23		
Hypertensive Status		1.02–2.42	0.042		1.04–1.66	0.021
Yes	1.57			1.32		
No	1			1		
Diabetes Status		1.38–4.69	0.003		0.84–1.45	0.496
Yes	2.54			1.1		
No	1			1		
Previous History of Stroke		0.38–20.56	0.317		1.01–5.00	0.047
Yes	2.78			2.25		
No	1			1		
Previous History of Heart Attack		0.73–5.76	0.173			
Yes	2.05					
No	1					

*OR* Odds ratio, *CI* Confidence Interval

This table also shows that study respondents aged 45 years and above are at 1.68 times higher odds (95% CI: 1.39–2.03, *p* < 0.001) of having good recognition of stroke symptoms compared to those aged less than 45 years old. Being a Malay is 1.49 times more likely to recognize stroke symptoms as compared to non-Malays (95% CI: 1.24–1.79, *p* < 0.001). Besides that, those who are hypertensive are 1.32 times more likely to be equipped with good recognition of stroke symptoms compared to non-hypertensives (95% CI: 1.04–1.66, *p* = 0.021). In addition, those who had a previous history of stroke are 2.25 times more likely to have good recognition of stroke symptoms (95% CI: 1.01–5.00, *p* = 0.047).

## Discussion

Our study showed that the knowledge including the correct recognition of stroke symptoms among the respondents were good as there were at least more than 60% of the study respondents who answered correctly for each of the questions. Our study also demonstrated that 83% of the respondents know what organs that are affected by stroke. Our results seem to show higher knowledge about strokes compared to studies done in other developing countries such as India and Oman where more than half of their study respondents were unaware of which organ is affected by stroke [7, 14]. The differences in the findings may be explained by the fact that Alshafee et al. carried out their study among two semi-urban village residents [14] while the study subjects in Pandian et al. study were relatives of patients without a history of stroke who were attending the outpatient department of a hospital [7]. The participants in our study came from screening done mainly at urban community clinics, hospitals, community centres, family day events and charity run and possibly have better access and exposure to information on stroke at health fairs, public health education events.

This possibly explains why our respondents have a better knowledge regarding stroke and this should augur well in term of stroke prevention activities.

Surprisingly, 16% of our respondents answered that heart was the organ affected by stroke and this finding was similar to a local study conducted by Sowtali et al. among stroke patients admitted to a tertiary hospital in Malaysia, in which 10.5% of the respondents were unable to differentiate between stroke and heart attack [6], indicating an important knowledge gap.

In our study, we found that up to 74.3% of the study respondents were able to identify all 5 symptoms of stroke correctly indicating that the level of knowledge on this aspect is high and this hopefully can translate into better practices in regards to stroke. This is relatively much higher than the local study done in 2014 among the general public in two different urban populations who attended 2 community-based events in Malaysia which only 35% of study respondents were able to identify 5 symptoms of stroke and above correctly [12]. Our results were comparably higher than a study done among relatives of patients without history of stroke who were attending the outpatient department of a hospital in India in which only 6.2% of the study respondents were able to identify all symptoms of stroke correctly [7]. In China, only 3.3% of patients from Hunan province with a previous history of stroke or transient ischemic attacks in China were able to identify all warning signs of stroke accurately [9]. When compared to developed countries, it was found that our awareness of stroke symptoms was also higher [15, 16]. The studies by Pancioli et al. as well as Sung Yoon et al. in developed countries were conducted among the general public using telephone interview in which the participants were asked about risk factors and symptoms of stroke [15, 16]. In detail, the study in Greater Cincinnati, Ohio, metropolitan area by Pancioli et al., reported that 57% of the respondents correctly listed at least 1 of the 5 established stroke warning signs, while 68% correctly listed at least 1 of the established stroke risk factors [15]. Meanwhile, the study by Sung et al. on an urban community in Newcastle, Australia, found that 76.2% respondents correctly listed ≥1 established stroke risk factor, but only 49.8% respondents correctly listed ≥1 warning sig n[16]. Recognition of stroke symptoms is very crucial to enable earlier access to medical care as it will definitely improve the outcome of stroke as reported in the literatur e[9, 17].

Among all 5 symptoms, the most commonly identified symptom is slurred speech which was answered correctly by 92.1% of the total study respondents. This was similar to a study done by Rowe et al. in the United States of America, in which up to 93% of the study respondents in that study recognized that slurred speech as one of the most common symptoms of stroke [18]. Meanwhile, this is in contrast with studies done in neighbouring countries, namely China and Nepal in which more than 60% of the study respondents which included young adults as well as older adults who identified sudden weakness of limbs as the most common symptoms of stroke [9, 10]. Therefore, health campaigns on stroke should emphasised these common 5 symptoms of stroke to enable early recognition of the onset of stroke and the need to present to hospital early.

In our study, it was found that respondents aged 45 years and above are more likely to recognize all 5 common symptoms of stroke and have good knowledge on the actions to be taken during stroke as compared to those who are aged less than 45 years. This may be due to the fact that elderly people are more likely to have multiple comorbidities especially hypertension and diabetes, which are commonly associated with stroke. Therefore, clinicians must have emphasized stroke as the possible complications of the above comorbidities. Subsequently, this will help people from all age groups, especially in the older adults to increase awareness towards the risk factors of stroke as noted in ta previous study [19]. However, this is in contrast with a systematic review among the general public with or without history of stroke that showed that generally older age group of people have poorer knowledge of stroke and are less likely to call for an ambulance whenever they experience symptoms of stroke [20]. The difference in the findings could be explained by two facts, one that the later study was a systematic review involving many different populations and secondly it was published nearly a decade ago where people had a shorter life expectancy and lesser opportunities for consultations with physician. In Malaysia, for example, it was only after the year 2010, that most clinical practice guidelines on important health conditions such as diabetes, stroke and hypertension were published and this has given rise to more evidence-based consultations by the physicians to their patients.

In terms of knowledge regarding limitations in performing the activities of daily living due to stroke, majority of the respondents agreed that 4 core activities will be affected i.e. dressing yourself, able to work, able to drive and able to go to the toilet without assistance. These findings are supported by the fact that most stroke survivors will have problems with dressing, walking, eating, bed transfer and bathing post-stroke [21]. This is also consistent with previous knowledge that certain activities of daily living such as bathing, dressing, ambulating and toileting are affected as one ages, what more if one has a condition that causes severe disability such as stroke [22].

The most important and accurate action to be undertaken in the event of an acute stroke is that the affected individuals must be able to recognise it and go to hospital within the golden period of 4.5 h and indeed 74.2% of the respondents did answered that, with majority of the respondents(82.1%) also saying that they would go to hospital on the same day. A third of the individuals (31.6%) responded that they may also seek for alternative therapy e.g. acupuncture. Just under half (47%) of the respondents would seek the combination of hospital and traditional treatment. This is consistent with a previous study in Malaysia where two thirds of stroke survivors (66%) reported using some form of complementary and alternative medication (CAM) as an adjunct to the physician prescribed medications [23]. This important finding indicates that the use of CAM should be explored in all patients with stroke as there potentially may be serious drug to drug interactions.

Malay ethnicity was found to be a determinant of both the good knowledge on the actions to be taken during stroke and recognition of stroke symptoms. This is consistent with a study done in Malaysia which showed that Malays tend to have good knowledge of stroke risk factors [6]. This could be due to the fact that Malay respondents tend to consult physicians when facing any health problem and other ethnicities were more likely engage into self-medication as reported by Dawood et al., 2017 which was a cross sectional study looking at health behaviour and self-medication practices among the general public [24]. Thus, more efforts must be directed to those who are of other ethnicities in order to improve their level of knowledge in order to equip them with good practice of stroke whenever an attack strikes them.

It is surprising that educational level was shown to have no association to both good knowledge on the actions to be taken during stroke and recognition of stroke symptoms in our study. This is most likely due to the fact that we have a big number of missing data that could affected the possibility of having any significant association. However, many studies had been demonstrating that higher level of education has been associated with better knowledge of stroke based on studies mainly done on relatives of patients with or without history of stroke attending hospitals [6, 14, 25]. However, a study done in Australia has revealed that despite the presence of a significant association between the level of education and knowledge of stroke, education does not confer any advantage in term of taking proper response towards stroke warning signs [16]. Nevertheless, further studies need to be conducted to confirm this association and educational strategies need to be carried out targeting those with lower educational qualification to increase the awareness of stroke in order to foster correct emergency steps to be taken especially whenever an attack occur.

A Few studies done in America, India and China showed that hypertension is the most commonly identified risk factors of stroke as identified by the adults in cross-sectional study involving stroke survivors and also in review articles [5, 9, 26]. Meanwhile, a previous study done in Malaysia also showed a similar result in which more than 80% of the study respondents identified hypertension as the main risk factor of stroke [6]. Our study demonstrated that respondents who have hypertension are more likely to have good knowledge on the actions to be taken during stroke as well as recognition of stroke symptoms. Those who have hypertension will have higher level of concern on their future risk of getting stroke compared to non-hypertensives. As a result, this will encourage self-driven initiatives in acquiring more knowledge regarding stroke either through surrounding family members, friends or social media. Besides that, patients with hypertension would also receive more information regarding stroke from healthcare providers, as compared to those who are not hypertensive.

Being a diabetic was also shown to be associated with good knowledge on the actions to be taken during stroke. The possible reason could be due to the fact that those with underlying diabetes mellitus were at 4 times higher risk of having stroke, recurrent stroke, longer hospital stay, higher functional disabilities, stroke-related dementia and also higher mortality rate), therefore making them more vigilant and aware towards the symptoms of stroke [27–30].

In our study, patients who had a previous history of stroke are more likely to have good knowledge of stroke and able to recognize all five common symptoms of stroke. This finding is expected and is consistent with previous studies on patients with a history of coronary heart disease (high risk) and general public (low risk) [16, 31]. This clearly showed the importance of those who have a past history of stroke to be well-equipped with information regarding stroke as good recognition of stroke symptoms ensures effective and optimal management. Despite our study showed relatively good result on the level of knowledge of symptoms recognition, there are still room for improvement. Therefore, educational interventions need to be targeted to those who had no previous history of stroke to enhance their basic knowledge on stroke.

Our study showed that non-smokers have better knowledge on the actions to be taken during stroke of stroke compared to those who were smokers. It was established in literature that smokers are less likely to engage in health-seeking behaviours in studies focusing on smokers [32–35]. This is worrisome as the relationship between number of cigarettes smoked and the risk of stroke has been well-established in previous literature. It was stated that the risk of stroke among smokers is 6 times as compared to those who are non-smokers [36]. Despite our study showing that smoking status is not a determinant of good knowledge of stroke, it does not negate the needs of implementing educational activities to raise public awareness targeting smokers regarding the important topic of stroke and the importance of smoking cessation in reducing the risk of stroke. This is especially imperative as those who had no knowledge of the risk factors of stroke are less likely to engage themselves in behavioural pattern consistent with stroke prevention practices, as shown in a study on, smoking cessation conducted through a telephone survey among the general population [37]. Therefore, physician should spend some time to adequately counsel smoker to quit smoking due to an inherent risk of causing a potentially life changing stroke.

As stroke is a leading cause of death in Malaysia, prevention of stroke is absolutely important. This can enforce by ensuring that risk factors commonly associated with stroke such as hypertension, dyslipidaemia, atrial fibrillation, diabetes mellitus and cigarette smoking are actively tackled and managed appropriately during consultations [1]. In addition, to prevent the recurrence of stroke, the treating physician should ensure that the patients are optimized on their risk factor’s management as well as having good adherence towards their medications [1].

In terms of knowledge regarding the limitation of activities of daily living due to stroke, majority of the respondents agree that 4 core activities will be affected i.e. dressing yourself, able to work, able to drive and able to go to the toilet without assistance. This agrees well with the fact that most stroke survivors will have problems with dressing, walking, eating, bed transfer and bathing post-stroke as noted in a long-term prospective cohort study [21]. This is also consistent with previous knowledge that certain activities of daily living such as bathing, dressing, ambulating and toileting are affected as one ages, what more if one has a condition that causes severe disability such as stroke [22].

## Strengths and limitations

First of all, studies focusing on exploration of knowledge on the actions to be taken during stroke and recognition of stroke symptoms have been limited in the literature, left alone studies done on Malaysia. This study will serve as a pivotal addition to the existing literature. This study was carried out in multiple centres with a big sample size. In addition, this study has not only identified socio-demographic factors, but also explored the medical co-morbidities which associated with good practice and recognition of stroke symptoms. What is definitely worrying is the fact that the more productive segment of population which are the young people as well as non-hypertensives and non-diabetics all have poor practice and recognition of stroke symptoms.

This study has some limitations; firstly, the results have many missing data as there is time constraint during the BP screening as there is rush of people in a short period of time where individuals rushed to have their BP done and individuals have not enough time to answer and the researchers also have no time to countercheck it. This may lead to miss out possible significant association especially for the education variable which has previously shown a strong association with the knowledge of the stroke. Nevertheless, despite the missing data, our study showed that 40% of the participants were having secondary school education level and this is consistent with the proportion of the nation’s population having the similar educational level [38]. Thus it may not influence the final result after all.

In addition, since this is a cross sectional study, the data of every respondent was only collected once within the specific period of time. By this means, only an association and not a causation relationship can be inferred in this study.

The representativeness of the sample and generalizability of the findings may also be a problem. It may be affected by the fact that participants were recruited from a blood pressure screening programme with the possibility that only persons that are health conscious or concerned about their health were willing to participate in the study. Hence, some groups of the community may have been underrepresented or overrepresented and therefore, the finding of still study may not be generalisable. However, we think this may not a big issue as the prevalence of hypertension among adult aged 18 and above in this study (32.4%) is similar to that of randomly selected national study (30.3%) [11, 39]. We inadvertently missed out the important question of “the presentation could be paresis of the mirror half of the body” as it is one of the questions that can test knowledge of recognition of the symptoms of stroke. In addition, important data such as the presence of diabetes, hypertension, and previous history of stroke or heart attack were subjectively obtained from the respondents and may not be entirely reliable data. Therefore, readers are advised to interpret the findings of this study cautiously.

## Conclusion

Our study demonstrated that Malaysians has an overall good knowledge regarding stroke particularly on the recognition of stroke symptoms and actions to be taken during stroke. Almost half of the study participants will opt for some form of CAM if they encounter stroke and this important finding should prompt the healthcare providers to explore the use of CAM in all patients with stroke as there may be potentially cause a drug to drug interactions. Older age, being a Malay, non-smokers, hypertensives and diabetics are determinants of right actions to be taken during stroke. Meanwhile, older age being a Malay, hypertensive and those who had a previous history of stroke are determinants of good recognition of stroke symptoms.

## Data Availability

Raw data can be made available on request to the corresponding authors.

## Abbreviations

CI: Confidence Interval
ISH: International Society of Hypertension
MMM17: May Measurement Month 2017
MREC: Medical Research Ethical Committee
NMRR: National Medical Research Register
OR: Odds Ratio
SPSS: Statistical package for social sciences
UMMC: University Malay Medical Centre
WHO: World Health Organization
